# Production of Bioactive Soluble Interleukin-15 in Complex with Interleukin-15 Receptor Alpha from a Conditionally-Replicating Oncolytic HSV-1

**DOI:** 10.1371/journal.pone.0081768

**Published:** 2013-11-27

**Authors:** David C. Gaston, Carl I. Odom, Li Li, James M. Markert, Justin C. Roth, Kevin A. Cassady, Richard J. Whitley, Jacqueline N. Parker

**Affiliations:** 1 Department of Cell, Developmental, and Integrative Biology, The University of Alabama at Birmingham, Birmingham, Alabama, United States of America; 2 Division of Infectious Diseases, Department of Pediatrics, The University of Alabama at Birmingham, Birmingham, Alabama, United States of America; 3 Division of Neurosurgery, Department of Surgery, The University of Alabama at Birmingham, Birmingham, Alabama, United States of America; 4 Department of Medicine, The University of Alabama at Birmingham, Birmingham, Alabama, United States of America; 5 School of Medicine, The University of Alabama at Birmingham, Birmingham, Alabama, United States of America; McMaster University, Canada

## Abstract

Oncolytic type-1 herpes simplex viruses (oHSVs) lacking the γ_1_34.5 neurovirulence gene are being evaluated for treatment of a variety of malignancies. oHSVs replicate within and directly kill permissive cancer cells. To augment their anti-tumor activity, oHSVs have been engineered to express immunostimulatory molecules, including cytokines, to elicit tumor-specific immune responses. Interleukin-15 (IL-15) holds potential as an immunotherapeutic cytokine because it has been demonstrated to promote both natural killer (NK) cell-mediated and CD8^+^ T cell-mediated cytotoxicity against cancer cells. The purpose of these studies was to engineer an oHSV producing bioactive IL-15. Two oHSVs were constructed encoding murine (m)IL-15 alone (J100) or with the mIL-15 receptor α (mIL-15Rα, J100D) to determine whether co-expression of these proteins is required for production of bioactive mIL-15 from oHSV. The following were demonstrated: i) both oHSVs retain replication competence and cytotoxicity in permissive tumor cell lines. ii) Enhanced production of mIL-15 was detected in cell lysates of neuro-2a cells following J100D infection as compared to J100 infection, suggesting that mIL-15Rα improved mIL-15 production. iii) Soluble mIL-15 in complex with mIL-15Rα was detected in supernates from J100D-infected, but not J100-infected, neuro-2a, GL261, and CT-2A cells. These cell lines vary in permissiveness to oHSV replication and cytotoxicity, demonstrating soluble mIL-15/IL-15Rα complex production from J100D was independent of direct oHSV effects. iv) The soluble mIL-15/IL-15Rα complex produced by J100D was bioactive, stimulating NK cells to proliferate and reduce the viability of syngeneic GL261 and CT-2A cells. v) J100 and J100D were aneurovirulent inasmuch as no neuropathologic effects were documented following direct inoculation into brains of CBA/J mice at up to 1x10^7^ plaque forming units. The production of mIL-15/mIL-15Rα from multiple tumor lines, as well as the lack of neurovirulence, renders J100D suitable for investigating the combined effects of oHSV and mIL-15/IL-15Rα in various cancer models.

## Introduction

 Oncolytic type-1 herpes simplex viruses (oHSVs) deleted of the diploid γ_1_34.5 gene are being actively investigated as a therapy against multiple forms of cancer. oHSVs have been investigated in Phase I or II clinical trials for malignant gliomas, malignant melanoma, head and neck squamous cell carcinoma, and cutaneous metastases of varying cancers [1-10]. Independent Phase I and Phase Ib studies have established the safety of administering oHSV directly to the central nervous system (CNS) of patients with malignant glioma [2,5]. Although wild-type HSV-1 infection in the CNS can result in devastating encephalitis, deletion of the diploid γ_1_34.5 neurovirulence gene renders the therapeutic oHSV safe even for treatment of malignancies arising in the brain due to the inability of the virus to replicate in nonmalignant, post-mitotic cells [11]. The cytotoxicity of γ_1_34.5-deleted oHSV is restricted to permissive tumor cells containing oncogenic mutations that complement the function of the γ_1_34.5 gene product [12].

Direct oHSV-mediated cytotoxicity and indirect stimulation of immune responses cooperate to enhance the anti-tumor effects of oHSV [13-15]. Accordingly, oHSVs have been engineered to express a variety of immunotherapeutic genes with the intent of stimulating cellular anti-tumor immune responses. In pre-clinical studies oHSV engineered to express the murine genes encoding interleukin-12 (IL-12), interleukin-4 (IL-4), chemokine (C-C) motif ligand 2 (CCL2), or human granulocyte-macrophage colony stimulating factor (GM-CSF) were reported to reduce tumor burden or improve survival of tumor bearing mice as compared to parental non-cytokine encoding oHSV [16-20]. Increased tumor infiltrating immune cells, including CD4^+^ and CD8^+^ T cells, NK cells, and macrophages were documented following administration of oHSVs encoding IL-4 and IL-12 as compared to non-cytokine encoding oHSVs [16,17,20]. Tumor bearing mice administered an oHSV encoding GM-CSF developed tumor-specific immune responses and were protected from re-challenge of tumor [19].

Interleukin-15 (IL-15) is an immunostimulatory cytokine that has received attention recently as a promising cancer immunotherapeutic agent [21,22]. The IL-15 cytokine/receptor signaling complex is composed of IL-15, IL-15 receptor alpha (IL-15Rα), IL-2/IL-15 receptor beta (IL-2/IL-15Rβ), and the common gamma chain (γ_C_) [23-25]. IL-15Rα binds IL-15 and presents the cytokine *in trans* to cells displaying the IL-2/IL-15Rβ and γ_C_ components of the receptor, such that IL-15Rα is not required on the responsive cell for signaling to occur [26]. IL-15 alone can stimulate responsive cells, but stimulation is significantly enhanced when in complex with IL-15Rα [27-31]. Co-expression of IL-15 and IL-15Rα results in formation of the IL-15/IL-15Rα complex [32]. IL-15Rα associates with IL-15 in the endoplasmic reticulum, after which the IL-15/IL-15Rα complex is glycosylated in the Golgi apparatus and trafficked to the cell surface [33]. The IL-15/IL-15Rα complex can be presented on the cell surface as well as released as a soluble complex [34]. Soluble IL-15/IL-15Rα complex is physiologically relevant, as the majority of soluble IL-15 in human blood is in complex with IL-15Rα [35].

Interest in IL-15 as an immunotherapeutic agent is founded primarily on the ability of the cytokine to stimulate natural killer (NK) cells and CD8^+^ T cells. IL-15 activates NK cells to become cytotoxic, promotes NK cell survival and proliferation, and enhances production of inflammatory cytokines [36-38]. IL-15 also stimulates CD8^+^ T cell proliferation, enhances cytotoxicity, and promotes the development and maintenance of memory CD8^+^ T cells [24,39-41]. Preclinical studies in multiple tumor models have demonstrated therapeutic administration of IL-15 can reduce tumor burden and lengthen survival of tumor bearing animals. The anti-tumor effects of IL-15 have been documented following systemic administration of IL-15 in complex with soluble IL-15Rα, as well as engineering tumor cells to produce IL-15 alone in the local tumor microenvironment [29,42-46]. Additionally, gene therapy approaches delivering IL-15 DNA to tumors using plasmid vectors or engineered viruses have successfully reduced tumor burden [47,48]. In many of these studies decreased tumor burden and prolonged survival was abolished when mice were depleted of NK and/or CD8^+^ T cells, demonstrating these IL-15 responsive effector cells were providing the anti-tumor effects. The safety of intravenously delivered IL-15 was demonstrated in Rhesus macaques with no significant toxicity [49]. Multiple clinical trials evaluating systemically administered IL-15 as a therapeutic agent for treatment of cancers of varying origins are underway.

 The studies herein report the construction of an oHSV (J100D) producing murine (m)IL-15 in complex with mIL-15Rα (mIL-15/IL-15Rα). To achieve this, genes encoding both mIL-15 and mIL-15Rα were independently inserted into separate gene loci in a single oHSV backbone. J100D produces soluble mIL-15/IL-15Rα complex following infection of murine neuroblastoma and murine glioma cell lines with variable permissiveness to oHSV replication and killing. The soluble mIL-15/IL-15Rα complex is bioactive, as demonstrated by its ability to stimulate enriched murine splenic NK cells to survive and proliferate in culture as well as reduce the viability of syngeneic glioma cells. A control oHSV encoding mIL-15 alone (J100) replicated and killed permissive cells, but did not produce mIL-15/IL-15Rα complex. Both oHSVs were aneurovirulent when directly injected into the brains of mice. The construction of J100D, demonstrated to produce soluble, bioactive mIL-15/IL-15Rα complex enables the investigation of combinatorial anti-tumor approaches using oHSV and mIL-15/IL-15Rα in multiple cancer models.

## Methods

### Cells, Plasmids, and Viruses

Vero, Rabbit Skin Cells (RSC), Neuro-2a, and GL261 cell lines were obtained from the American Type Culture Collection (Manassas, VA). The CT-2A cell line was a kind gift from Thomas Seyfried, Ph.D. (Boston College, Boston, MA) [50]. The 4C8 cell line was a kind gift from Richard Pyles, Ph.D. (University of Texas Medical Branch at Galveston, Galveston, TX) [51]. Vero cells were maintained in complete Modified Eagle’s Medium (MEM) with 7% fetal bovine serum (FBS). RSC cells were maintained in Dulbecco’s Modified Eagle Medium (DMEM) with 5% FBS. Neuro-2a and 4C8 cells were maintained in complete DMEM/F12 with 7% FBS, whereas GL261 and CT-2A cells were maintained in complete DMEM with 7% FBS. During infection media was changed to contain 1% FBS, but replenished with media containing 7% FBS following infection. All cells were grown in tissue culture incubators at 37°C with 5% CO_2_.

Intronless mIL-15 and mIL-15Rα genes were amplified from the plasmids pORF9-mIL15 and pORF9-mIL15RAa (InvivoGen, San Diego, CA), respectively, using the primers F-AAACTAGTCGACCCTGAGATCACCGGTAGG and R-AAGGATCCTCTAGAAATAACAGAAACACGG for mIL-15 and F-AAAATCTAGACTGAGATCACCGGTCACC and R-AAAACTCGAGCTTAGGCTCCTGTGTCTTC for mIL-15Rα. The primers introduce novel restriction enzyme sites that allow subcloning into targeting vectors containing HSV-1 sequences. The novel restriction enzyme sites for the mIL-15 gene are SalI and XhoI, and those for the mIL-15Rα gene are XbaI and XhoI. The mIL-15 amplicon was first subcloned into the pCA13 plasmid containing the cytomegalovirus immediate early (CMV_IE_) promoter and the simian virus 40 (SV40) polyadenylation sequence (SV40pA) following restriction enzyme digestion of the mIL-15 amplicon with SalI and XhoI, digestion of the pCA13 plasmid with SpeI and XhoI, and ligation of resultant complementary sequences. The expression cassette was excised by digestion with BgIII, isolated by agarose gel electrophoresis, and subcloned into the targeting plasmid pCK1037 following BglII digestion of the plasmid. pCK1037 contains segments of the HSV-1 genes U_L_3 and U_L_4 to allow insertion of transgenic DNA into the intergenic region between the U_L_3 and U_L_4 genes. The mIL-15Rα amplicon was subcloned directly into the targeting plasmid pRB4878ssx, which contains the early growth response gene-1 (Egr-1) promoter, the hepatitis B virus polyadenylation sequence (HepBpA), and sequences flanking the HSV-1 γ_1_34.5 gene, by digestion of the mIL-15Rα amplicon and pRB4878ssx with SpeI and XhoI followed by ligation of complementary sequences. Plasmid construction was verified by restriction enzyme digest analysis. oHSV expressing mIL-15 with or without mIL-15Rα were sequentially constructed by the “bridge plasmid method” in RSC cells and verified by Southern blot analysis as has been previously described [16,17,52]. Wild-type HSV-1 (F) strain and the γ_1_34.5-deleted R3616 were kind gifts from Bernard Roizman, ScD (University of Chicago, Chicago, IL). Construction of the virus C101 has been previously described [53].

### Replication and oHSV Cytotoxicity

Neuro-2a, CT-2A, and GL261 cells were infected with a multiplicity of infection (MOI) of 0.1 for multi-step replication assays, as previously described [16]. oHSV-cytotoxicity was assessed by alamarBlue dye (Life Technologies, Carlsbad, CA) conversion following infection of cells at MOIs ranging from 0.03 to 100 as previously described [17,54]. Briefly, cells were seeded into 96 well plates in subconfluent monolayers. These cells were infected the following day with increasing half-log MOIs of the indicated oHSV in quadruplicate. The toxic dose at which 50% of cells were killed (TD_50_) was calculated 72 hours following infection by addition of alamarBlue dye and measuring dye conversion with a spectrophotometer after four hours.

### Western blot and ELISA

For Western blot, samples collected in SDS-containing lysis buffer were separated on a 12% SDS-PAGE gel, transferred to a nitrocellulose membrane, and probed with anti-IL-15 polyclonal rabbit primary antibody (H114, Catalog number sc-7889, Santa Cruz Biotechnology, Santa Cruz, CA) diluted 1:500. An HRP-conjugated anti-rabbit secondary antibody diluted 1:10,000 was used for detection. Membranes were stripped, blocked, and probed with an antibody against beta-actin as a loading control.

To quantify mIL-15/IL-15Rα complex, enzyme linked immunosorbent assay (ELISA) was used. Supernates were collected from 3 to 72 hours post infection (hpi) and frozen at -20°C. The mouse IL-15/IL-15R complex ELISA Ready-SET-Go! (Catalog number 88-7251, eBioscience, San Diego, CA) was used for the detection of mIL-15/IL-15Rα complex from supernates per manufacturer instructions.

### Animals

6 to 8 week old CBA/JCr, B6D2F1, and C57BL/6 mice were obtained from Frederick Cancer Research Center (Frederick, MD). Mice were housed in the Animal Care Facility in the Children’s Harbor Center, Birmingham, AL.

### Ethics Statement

All procedures involving mice were performed with oversight and approval by the University of Alabama at Birmingham Institutional Animal Care and Use Committee (UAB IACUC, Animal Project Number 08936). Mice undergoing surgical procedures were anesthetized with a mixture of ketamine and xylazine. All efforts were put forth to minimize suffering during or following surgery as well as upon sacrifice.

### Sample Preparation for Bioactivity Assays

Neuro-2a cells were mock infected or infected with J100, or J100D, at an MOI of 10. After infection, cells were re-fed with one-quarter of the standard volume of growth medium to increase cytokine concentration. The growth medium used to re-feed the cells contained 50 μM acyclovir to decrease virus in the supernates out of concern for observing effects attributable to infectious virus in subsequent assays. At 72 hpi the supernates were collected, frozen, thawed, and exposed to Neuro-2a cells in 100mm culture plates to further reduce residual virus in supernates. The mIL-15/IL-15Rα concentration was quantified using an ELISA specific for the mIL-15/IL-15Rα complex. Recombinant mIL-15/IL-15Rα (Catalog number 14-8152, eBioscience, San Diego, CA) was supplemented into mock-infected supernatant to produce positive control samples containing a final mIL-15/IL-15Rα complex concentration of 20 ng/mL. Supernates obtained from J100D infected cells were diluted with growth media such that the final mIL-15/IL-15Rα concentration equaled 20 ng/mL. The same volume of growth media used to dilute the supernates obtained from J100D infected cells was used to diluted supernates obtained from mock or J100 infected cells.

### NK Cell Enrichment and Proliferation

B6D2F1 mice were sacrificed according to UAB IACUC protocols and their spleens removed by aseptic technique. Splenocytes were obtained by physical disruption of the spleen and lysis of erythrocytes with 0.85% NH_4_Cl. NK cells were enriched with a negative-selection procedure (Catalog number MAGM210 MagCellect Mouse NK Cell Isolation Kit, R&D, Minneapolis, MN). Cells were stained with antibodies against CD49b (PE conjugated clone DX5, eBioscience, San Diego, CA) and CD8 (FITC conjugated, BD Biosciences, San Jose, CA) to assess enrichment. Enriched NK cells were stained with 4uM carboxyfluorescein succinimidyl ester (CFSE) and plated in a 96 well plate at a density of 1.5x10^5^ cells per well in 100 μL Roswell Park Memorial Institute (RPMI) 1640 media with 10% FBS and 50 μM acyclovir. 100 μL of supernatant prepared as described in the “sample preparation for bioactivity assays” section was overlaid to yield a final mIL-15/IL-15Rα concentration of 10 ng/mL in positive control and J100D sample wells. Cells were cultured up to 7 days. Cells were incubated with Fc block (BD Biosciences, San Jose, CA) prior to staining with a PE-conjugated NKp46 antibody (clone 29A1.4, BD Biosciences, San Jose, CA) for analysis by flow cytometry. Data was analyzed using FlowJo software (Tree Star Inc., Ashland, OR).

### NK Cell Mediated Viability Reduction Assay

4C8, GL261, or CT-2A cells were seeded into separate wells of 96 well plates at 5x10^3^ cells/well for 4C8 cells or 3x10^4^ cells/well for GL261 and CT-2A cells. NK cells from syngeneic mice (B6D2F1 for 4C8, C57Bl/6 for GL261 and CT-2A) were enriched and overlaid onto the glioma cells at the indicated ratios. 4C8, GL261, or CT-2A cells in matched wells were overlaid with media lacking NK cells. Supernatant prepared as described in the “sample preparation for bioactivity assays” section was added to all wells. Wells containing supernatant obtained from J100D infected cells contained a final mIL-15/IL-15Rα concentration of 10ng/mL. After co-culture for 3 days, the MTT reagent was added to all wells. Colorimetric conversion after four hours was measured by a spectrophotometer. The percent of viable cells in each experimental condition (media from mock, J100, or J100D infected cells) was calculated by dividing averaged optical density measurements of tumor cells with NK cells by averaged optical density measurements of tumor cells without NK cells.

### Neurotoxicity

Dilutions of wild-type HSV-1 (F) strain, R3616, J100 and J100D were prepared in sterile saline. Dilutions of J100 and J100D ranging from 3x10^4^ to 1x10^7^ plaque forming units (pfu) were stereotactically delivered intracranially in 5μL/mouse to CBA/JCr mice as previously described [55]. 1x10^5^ pfu of HSV-1 (F) strain and 3x10^6^ pfu of R3616 were delivered to two cohorts as positive and negative controls, respectively. Each virus dilution cohort contained 5 mice. Mice were monitored daily and euthanized upon development of neuropathologic signs.

### Statistical analysis

 Statistical significance was calculated using unpaired t tests assuming equal standard deviation with Prism software (GraphPad, San Diego, CA). *p* values less than 0.05 were considered statistically significant.

## Results

### Construction of recombinant γ_1_34.5-deleted oHSVs encoding mIL-15 with and without mIL-15Rα

**Figure 1:**
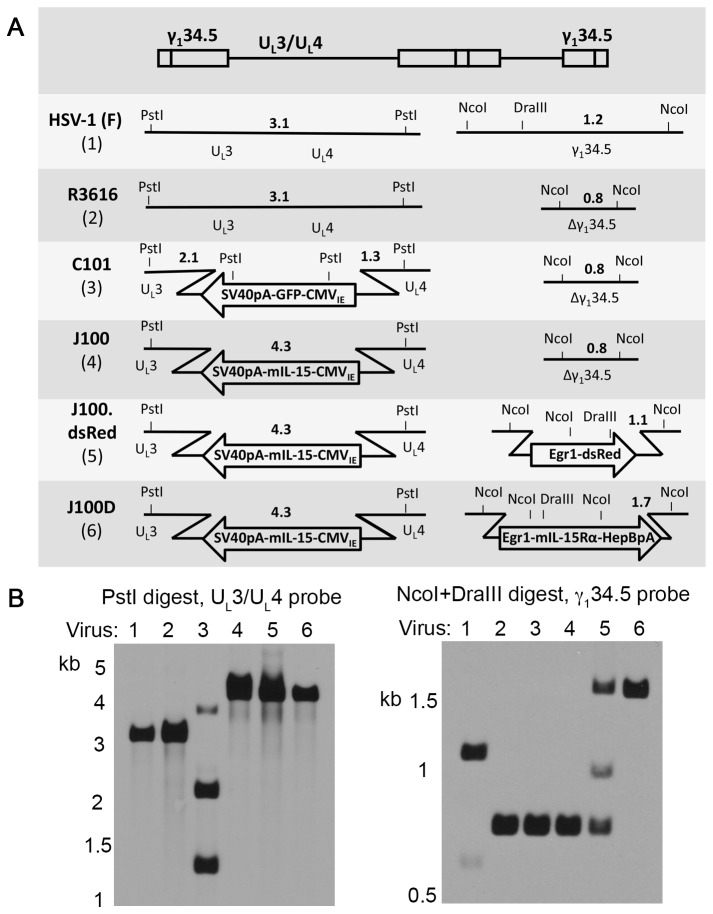
Construction of oHSVs encoding mIL-15 (J100) or mIL-15 and mIL-15Rα (J100D). mIL-15 and mIL-15Rα sequences were engineered into oHSV in the U_L_3/U_L_4 or γ_1_34.5 regions by homologous recombination. Recombination replaces green fluorescent protein (GFP) or dsRed genes, allowing negative color selection for candidate viruses. A) Schematic of engineered viruses demonstrating the derivation of J100 and J100D. The topmost illustration indicates the relative position of the genetic loci in the HSV-1 genome and is not drawn to scale. Diagnostic restriction enzyme sites are designated by PstI, NcoI, and DraIII. Bold numerals indicate predicted band sizes on Southern blot analysis with appropriate probes. B) Southern blots verifying virus construction and purity of J100 and J100D following restriction enzyme digestion of viral DNA with PstI for transgenes engineered into the U_L_3/U_L_4 intergenic region (GFP or mIL-15) or NcoI and DraIII for transgenes engineered into the γ_1_34.5 loci (dsRed or mIL-15Rα). Lane numbers correspond to the numbers beneath the virus names in A. Insertion of the mIL-15 and mIL-15Rα expression cassettes into the indicated loci was also verified by sequencing. CMV_IE_ – human cytomegalovirus immediate early promoter; SV40pA – simian virus 40 polyadenylation sequence; Egr1 – early growth response gene-1 promoter; HepBpA – hepatitis B virus polyadenylation sequence.

mIL-15 and mIL-15Rα genes were subcloned into plasmids containing homologous flanking sequences of the HSV-1 U_L_3/U_L_4 or γ_1_34.5 genes, respectively. Using these plasmids, oHSVs encoding mIL-15 alone or encoding mIL-15 and mIL-15Rα were sequentially constructed through homologous recombination (Figure 1A). The U_L_3/U_L_4-targeting mIL-15 plasmid was used to replace the green fluorescent protein (GFP) gene of the parental oHSV C101 to construct J100. The constitutively active CMV_IE_ promoter drives expression of the mIL-15 gene and the mRNA is stabilized by the SV40 polyadenylation sequence. A γ_1_34.5-targeting plasmid (pCK1136) was used to insert the dsRed gene into the diploid γ_1_34.5 locus of J100 to construct J100.dsRed. The γ_1_34.5-targeting mIL-15Rα plasmid was used to replace the dsRed gene of J100.dsRed to construct J100D. Expression of the mIL-15Rα gene is driven by the constitutively active Egr-1 promoter and the mRNA is stabilized by the HepB polyadenylation sequence. Virus construction was verified by Southern blot (Figure 1B). All major bands are present as expected. Minor bands present in J100.dsRed signify a mixed population of viruses with and without the dsRed expression cassette. These bands were resolved upon construction of J100D and indicate a pure virus population. Incorporation of the mIL-15Rα expression cassette into J100D was also verified by sequencing (not shown). As J100 was used to sequentially construct J100D, both oHSVs contain the same mIL-15 expression cassette in the U_L_3/U_L_4 intergenic region. The mIL-15 genes and portions of the CMV_IE_ promoters in J100 and J100D were sequenced to ensure no mutations had arisen during construction. The sequenced regions were identical, and sequencing verified that the mIL-15 gene encoded by J100 and J100D contains the murine IL-15 long signal peptide (LSP) (not shown). 

### J100 and J100D are replication competent and cytotoxic to permissive tumor cell lines

**Figure 2:**
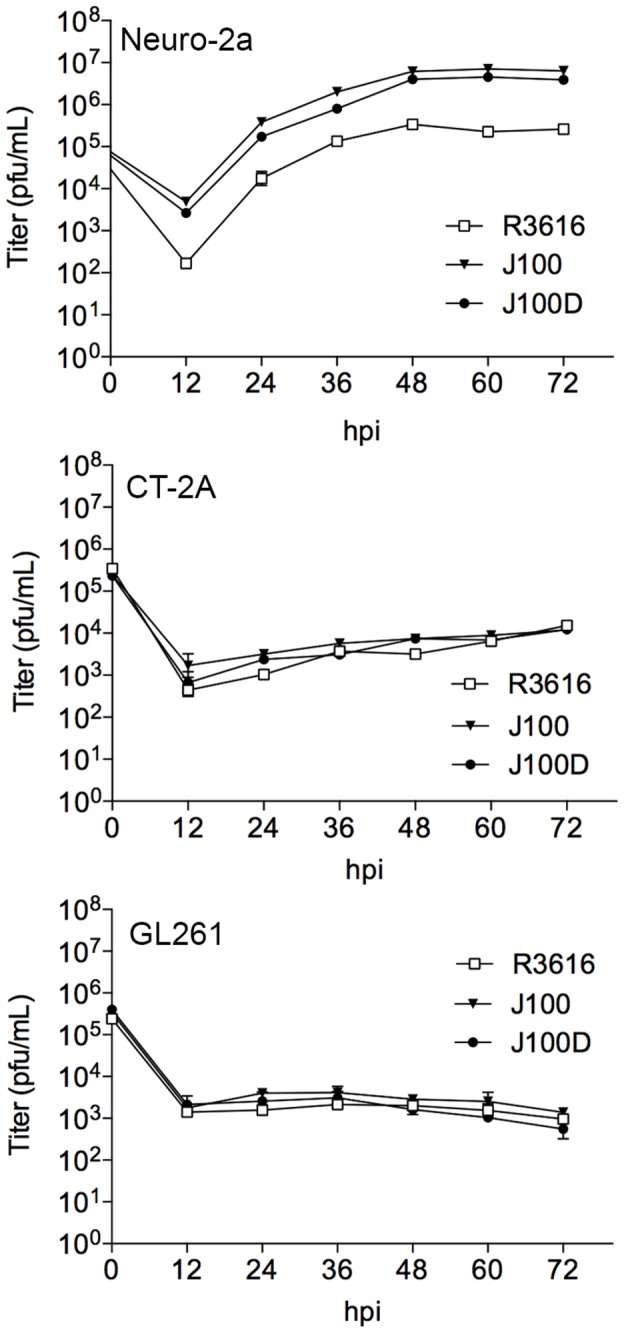
Replication of J100 and J100D in Neuro-2a, but not GL261 or CT-2A cell lines. In these multi-step replication assays, cells are infected at a multiplicity of infection (MOI) of 0.1 to allow multiple rounds of replication. The concentration of cellular-associated virus at each indicated timepoint was determined by limiting dilution plaque assay. Neuro-2a cells are permissive to oHSV replication as demonstrated by the amplification of infectious viral particles above the initial titer at infection. GL261 and CT-2A cells do not support replication of oHSVs. Individual data points represent the mean ± SD of triplicate samples. pfu – plaque forming units; hpi – hours post infection.

 Conditional replication competence is a primary attribute of oHSV [11,12]. The replicative abilities of J100 and J100D were assessed in a panel of tumor cell lines in analyses allowing multiple rounds of viral replication (Figure 2). J100 and J100D demonstrated similar replication efficiency in the Neuro-2a murine neuroblastoma line derived from A/J mice. This permissive tumor line allows abundant amplification of infectious progeny to titers multiple orders of magnitude above the input MOI. Although J100 and J100D replicated with greater efficiency than the parental virus R3616, this could be due to a lower input MOI or the previously reported low replicative capacity of R3616 [16]. Regardless, J100 and J100D replicate with nearly identical kinetics in Neuro-2a cells. R3616, J100, and J100D replicated poorly in the CT-2A and GL261 murine glioma cell lines derived from C57Bl/6 mice. Following an initial decrease in viral titer due to infection, the oHSVs were incapable of amplification similar to that observed in the Neuro-2a cell line. The titer of recovered virus did not increase beyond the infection titer at any timepoint in GL261 or CT-2A cells. Thus, the replicative abilities of J100 and J100D are equivalent although the viruses do not amplify efficiently in the tumor lines derived from C57Bl/6 mice.

 J100 and J100D cytotoxicity was evaluated using the alamarBlue assay in Neuro-2a, CT-2A, and GL261 cell lines to determine if the newly constructed viruses differed in direct cytotoxic effects (Table 1). J100 and J100D killed Neuro-2a and CT-2A cells, but not GL261 cells. The TD_50_ for GL261 cells following infection with J100 or J100D is greater than MOI 100, the highest MOI evaluated in these studies. Interestingly, the TD_50_ following J100D infection in Neuro-2a cells is significantly higher than the TD_50_ following J100 infection. The biologic significance of this difference remains to be determined.

**Table 1:** Cytotoxicity of J100 and J100D are similar yet vary per cell line.

	J100	J100D
Neuro-2a	4.38 ± 0.83	7.83 ± 1.28 *
CT-2A	5.57 ± 2.48	5.63 ± 2.72
GL261	>100	>100

For each half-log MOI increase from 0.03 to 100, cells were infected in quadruplicate and TD_50_ calculated by alamarBlue dye conversion at 72 hpi. The TD_50_ is the input MOI at which 50% of cells are killed by 72 hpi. The TD_50_ ± SD is presented, and the results are representative of two independent experiments. The TD_50_ for GL261 cells was not reached up to the MOI of 100. The TD_50_ in Neuro-2a cells is significantly higher for J100D as compared to J100 (* p = 0.004).

The replication and cytotoxicity results presented in Table 1 and Figure 2 demonstrate two important points: 1) that the effects of J100 and J100D are grossly similar, and 2) that the effects differ by individual cell line and not by the mouse strain of cell line origin. J100 and J100D both kill and replicate exponentially within Neuro-2a cells derived from A/J mice, only kill but do not replicate exponentially within CT-2A cells derived from C57Bl/6 mice, and neither kill nor replicate exponentially within GL261 cells derived from C57Bl/6 mice.

### J100D infection produces soluble mIL-15 in complex with mIL-15Rα from tumor cell lines with varying permissiveness to oHSV replication and killing

**Figure 3:**
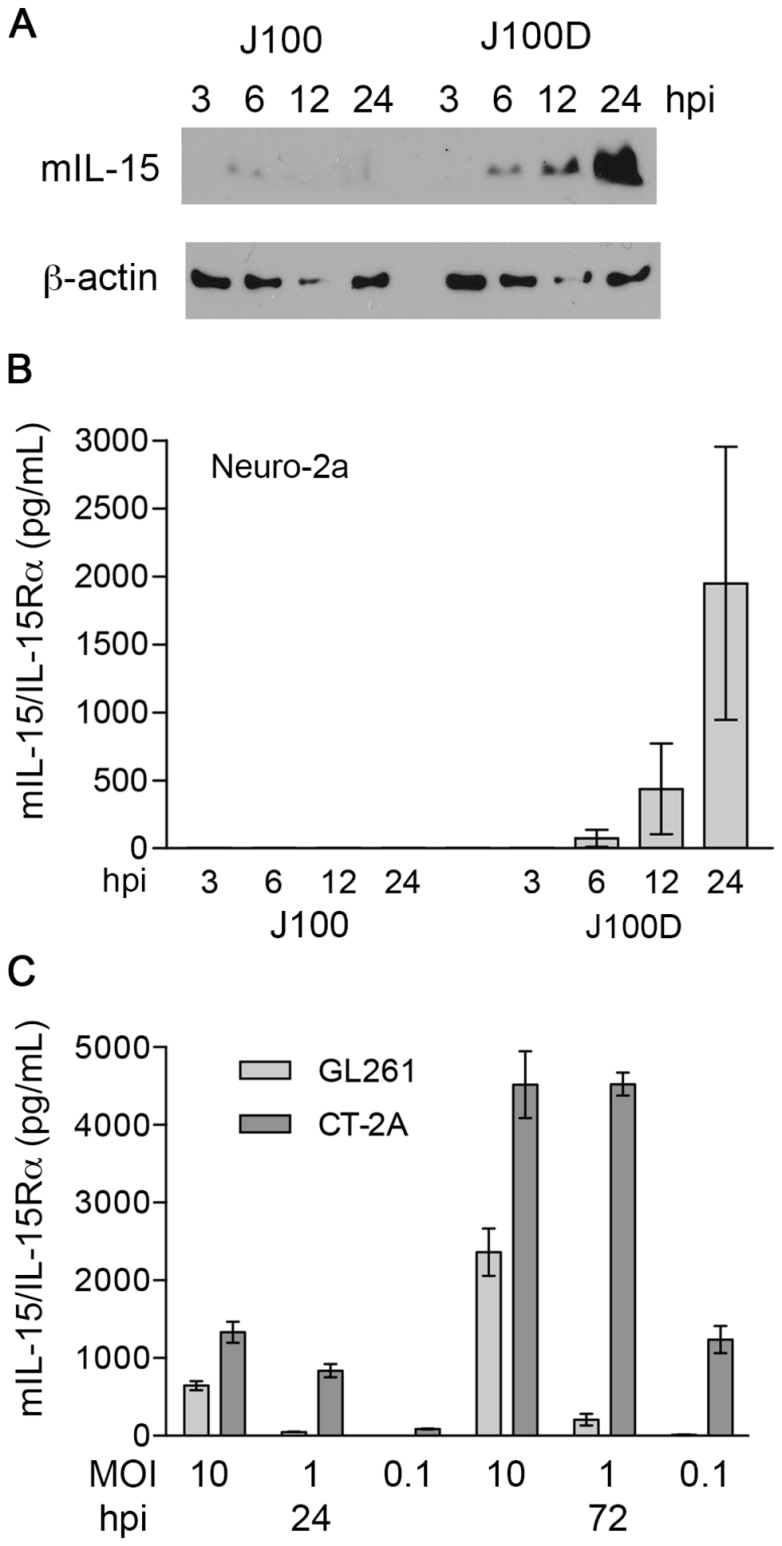
J100D expression of mIL-15 and mIL-15Rα increases mIL-15 production and results in soluble mIL-15/IL-15Rα complex. A) Western blot demonstrating mIL-15 production in Neuro-2a cells infected by J100 or J100D at a MOI of 10. B) ELISA demonstrating the presence of mIL-15/IL-15Rα complex in the supernatant of Neuro-2a cells infected by J100D, but not J100, at an MOI of 10. C) Quantification of mIL-15/IL-15Rα concentration in the supernates of J100D infected GL261 or CT-2A cells at the indicated hpi and MOIs. No mIL-15/IL-15Rα was detected in the supernates of J100 infected GL261 or CT-2A cells at any timepoint or MOI (not shown). The concentration of mIL-15/IL-15Rα in the supernates of CT-2A cells infected by J100D is significantly higher than the concentration in the supernates of infected GL261 cells at all MOIs and timepoints analyzed (*p* < 0.05).

 Production of mIL-15 from J100 and J100D was assessed in lysates of Neuro-2a cells infected at an MOI of 10 by Western blot (Figure 3A). Although a faint mIL-15 signal is present in the lysate sample from cells infected with J100 beginning at 6 hpi, this was not a reproducible finding (data not shown). However, production of mIL-15 protein following J100D infection was qualitatively greater than production following J100 infection at 6, 12, and 24 hpi. Equivalent sample loading per timepoint can be assessed by comparing β-actin staining of lysates from J100 and J100D infected cells at individual timepoints. J100 infection may produce mIL-15 protein, but mIL-15 protein production is greater following J100D infection. 

Supernatant samples from J100 and J100D infected Neuro-2a cells were assayed for soluble mIL-15/IL-15Rα complex using an ELISA specific for the mIL-15/IL-15Rα complex, but not mIL-15 or mIL-15Rα alone (Figure 3B). Soluble mIL-15/IL-15Rα complex was detected only in the supernates of J100D-infected cells, and increased in concentration from 6 to 24 hpi. Additionally, no mIL-15 was detected in the supernates of J100 infected Neuro-2a cells by a commercially available mIL-15 ELISA (data not shown). As the mIL-15 and mIL-15Rα genes encoded by J100D are expressed from constitutively active promoters, Neuro-2a cells were infected with J100D in the presence of the HSV-1 replication inhibitor acyclovir to ensure soluble mIL-15/IL-15Rα was produced in the absence of viral replication. No significant differences were observed in soluble mIL-15/IL-15Rα concentrations in the presence or absence of acyclovir, yet significantly decreased titers of virus were detected in the supernates when acyclovir was included in the culture media (Figure S1).

To determine if J100D infection resulted in soluble mIL-15/IL-15Rα complex production from poorly permissive tumor cell lines, mIL-15/IL-15Rα complex was quantified in the supernates of J100D infected GL261 and CT-2A cells. Soluble mIL-15/IL-15Rα complex increased in concentration from 24 to 72 hpi and was MOI dependent in both cell lines (Figure 3C). CT-2A cells produced significantly higher concentrations of the mIL-15/IL-15Rα complex as compared to GL261 cells at all MOIs and timepoints (*p* < 0.05). No mIL-15/IL-15Rα complex was detected in supernates of J100 infected GL261 or CT-2A cells.

### J100D-produced mIL-15/mIL-15Ra complex is bioactive

**Figure 4:**
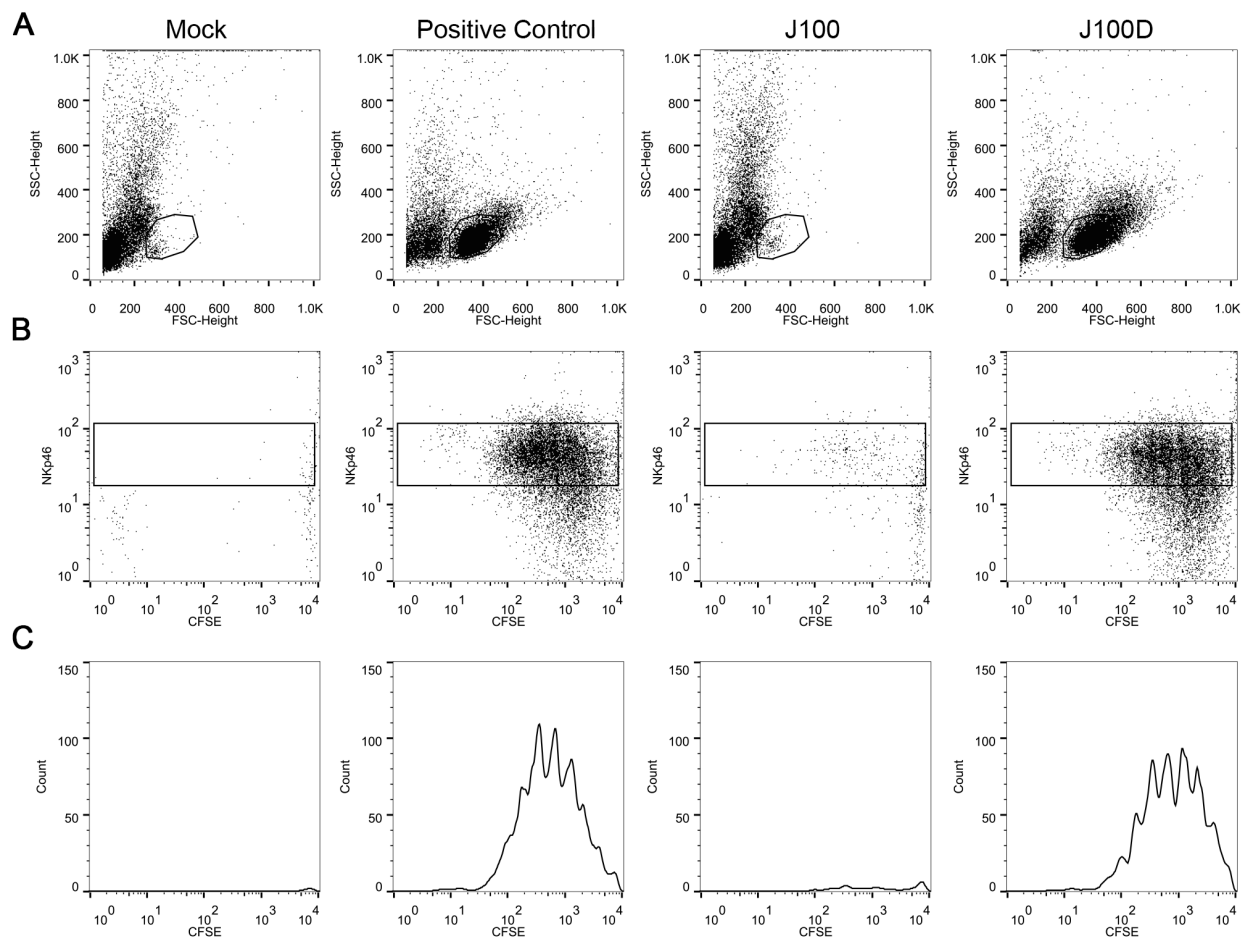
J100D-produced mIL-15/IL-15Rα stimulates survival and proliferation of enriched NK cells. Enriched NK cells were stained with CFSE and cultured in the presence of supernatant obtained from cells either mock infected or infected with the indicated viruses. Recombinant mIL-15/IL-15Rα complex was added to mock supernatant at 10ng/mL for the positive control, and the J100D sample was diluted to contain a final mIL-15/IL-15Rα complex concentration of 10ng/mL. Cells were cultured for 7 days prior to analysis. A) Forward vs. side scatter plots indicating survival of a population when mIL-15/IL-15Rα is present in the culture media. B - C) NKp46 staining and proliferation (as indicated by CFSE dilution) of cells present within the gate shown in A. Histograms presented in panel C are derived from the box gates in panel B. Data is representative of three independent experiments.

After determining that J100D produced soluble mIL-15/IL-15Rα complex, *in vitro* functional assays with enriched murine splenic natural killer (NK) cells were used to investigate the bioactivity of the complex. The release of mIL-15/IL-15Rα from J100D infected cells allows investigation of the bioactivity of the complex by adding conditioned supernatant from J100D infected cells into the culture media of NK cells. The unique ability of IL-15 to activate NK cells without the aid of other cytokines or growth factors facilitates investigation of the bioactivity of J100D-produced mIL-15/IL-15Rα. NK cells were enriched from the spleens of mice using a negative selection method to diminish the possibility of non-specific activation (Figure S2). Culturing enriched NK cells in the presence of mIL-15/IL-15Rα (10 ng/mL) produced by J100D resulted in a population of cells that stained positively for the NK cell marker NKp46 and had proliferated as indicated by CFSE dilution (Figure 4). A similar population of NKp46-positive, proliferating cells was observed upon culturing enriched NK cells with recombinant mIL-15/IL-15Rα at a concentration of 10 ng/mL. Enriched NK cells cultured with conditioned supernates from mock or J100 infected cells in which mIL-15/IL-15Rα was not detected by ELISA neither survived nor proliferated in culture.

The ability of J100D produced mIL-15/IL-15Rα complex to stimulate NK cell cytotoxicity was assessed using a viability reduction assay. The murine tumor cell lines 4C8, GL261, and CT-2A were plated separately and overlaid with NK cells enriched from the spleens of syngeneic mice at varying effector:target ratios. Conditioned supernatant samples from mock, J100, or J100D infected cells were added to the co-cultures such that the final mIL-15/IL-15Rα concentration in the co-cultures containing J100D-conditioned supernatant was 10ng/mL. Residual virus was present in the conditioned supernatant following sample preparation but did not exceed an MOI of 0.001 in the assays. Although it is unlikely that cell killing was viral-mediated at this low MOI, acyclovir was included in the culture media as a precautionary measure. After 72 hours of co-culture, cell viability was quantified using colorimetric dye conversion.

**Figure 5:**
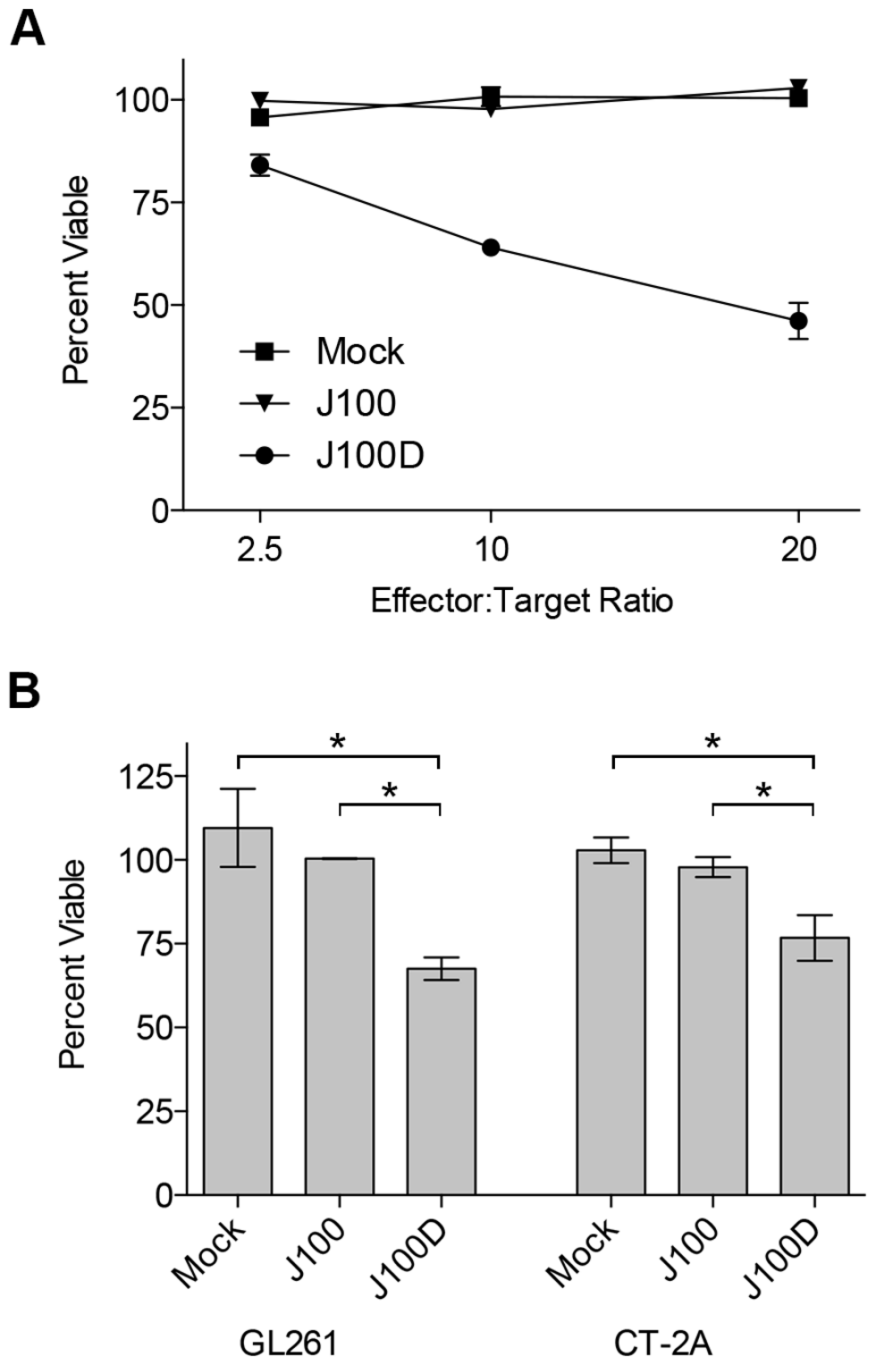
Reduced viability of tumor cells following co-culture with NK cells and J100D-produced mIL-15/IL-15Rα. Enriched murine NK cells were co-cultured for 3 days with syngeneic murine glioma cells. The cells were co-cultured in the presence of supernatant obtained from cells that were mock infected, or infected with J100, or J100D as described in the Methods. Cells co-cultured with supernatant derived from J100D infection were cultured in the presence of 10ng/mL of the J100D-produced mIL-15/IL-15Rα complex. Percent viability was assayed by colorimetric conversion of the MTT reagent after 72 hours of co-culture. A) 4C8 glioma targets cultured at increasing effector:target ratios with syngeneic B6D2F1 enriched NK cells. Data represents average values with standard deviations from triplicate samples. B) GL261 and CT-2A glioma targets cultured at an effector:target ratio of 2:1 with syngeneic C57Bl/6 enriched NK cells. Data represents the average value from two independent experiments performed with triplicate samples. Error bars indicate standard deviation. * *p* < 0.05 for J100D measurements compared separately to mock and J100 measurements.

4C8 cells were cultured with enriched B6D2F1 NK cells at effector:target ratios of 2.5:1, 10:1, and 20:1 (Figure 5A). The percent of viable cells decreased inversely with the effector:target ratios in cells co-cultured with media obtained from J100D-infeced cells, whereas no viability reduction occurred in cells co-cultured with media obtained from mock or J100-infected cells. No reduction in viability was measured even at the 20:1 effector target ratio in cells co-cultured with media obtained from mock or J100-infected cells. GL261 and CT-2A cells were cultured with enriched C57Bl/6 NK cells only at an effector:target ratio of 2:1 (Figure 5B). The percent viable GL261 and CT-2A cells were significantly reduced in the presence of supernates containing J100D-produced mIL-15/IL-15Rα as compared to co-culture with supernates obtained from mock or J100 infected cells. No difference in the percent of viable GL261 or CT-2A cells was observed in co-culture with mock or J100 conditioned supernates.

### J100 and J100D are aneurovirulent following direct intracranial injection in mice

Although deletion of γ_1_34.5 renders oHSV aneurovirulent, production of immunostimulatory transgenes from engineered oHSV could conceivably restore virulence. Neurovirulence was assessed by injecting increasing amounts of virus into the right cerebral hemispheres of CBA/JCr mice, a strain highly sensitive to the neuropathologic effects of HSV-1 (Table 2). All mice administered wild-type HSV-1 (F) strain as a positive control were euthanized due to weight loss and neuropathologic signs a median of 5 days following injection of 3x10^4^ pfu. One mouse of five administered 3x10^6^ pfu R3616 as a negative control was euthanized 3 days following virus injection due to weight loss but without any other neuropathic indicators. In this instance, the euthanized mouse was the smallest of the cohort, weighing 14g at the time of R3616 administration whereas the average weight of the other four mice was 17.6 ± 0.3g. As weight loss can occur in mice following intracranial administration of aneurovirulent oHSV, the euthanized mouse lost enough weight to require euthanization per IACUC guidelines. As no other neuropathologic signs were noted in this mouse it is unlikely that R3616 contributed to the death through neurovirulence. It is also notable that the TD_50_ for R3616 was not reached at 3x10^6^ pfu, a dose one hundred times higher than the wild-type HSV-1 (F) dose administered to the positive control cohort. Importantly, no mice administered J100 or J100D demonstrated neuropathologic signs or required euthanization, even following administration of virus doses up to 1x10^7^ pfu.

**Table 2:** J100 and J100D are aneurovirulent following injection into brains of CBA/JCr mice.

	WT HSV-1 (F)	R3616	J100	J100D
3x104 pfu	5/5		0/5	0/5
1x105 pfu			0/5	0/5
3x105 pfu			0/5	0/5
1x106 pfu			0/5	0/5
3x106 pfu		1/5	0/5	0/5
1x107 pfu			0/5	0/5

J100 and J100D were administered directly into the brains of CBA/JCr mice using stereotactic intracranial injection in doses increasing by half-log increments in 5μL saline. Five mice were injected per dose for each virus. HSV-1 (F) strain was used as a positive control and injected only at the lowest dose. 3x10^6^ pfu of R3616, the parent of J100 and J100D, were injected as a negative control.

## Discussion

 These studies describe the construction and characterization of J100D, an oHSV producing bioactive soluble mIL-15 in complex with mIL-15Rα. This is the first report of an oHSV engineered to express IL-15 or IL-15Rα of any species origin. Other oncolytic viruses have been engineered to express IL-15 alone, including adenovirus, recombinant adeno-associated virus type 2, vesicular stomatitis virus, influenza A, and myxoma virus [48,56-60]. To date, adeno-associated virus type 8 is the only other reported oncolytic virus engineered to express IL-15 and IL-15Rα [61].

The mIL-15/IL-15Rα complex is not encoded as a single transcript in J100D. The two genes are separated in the viral genome and expressed by separate promoters (Figure 1). This is notable because a previously engineered oHSV encoding the heterodimeric cytokine IL-12 did so using a single promoter and an internal ribosomal entry site (IRES) to allow translation of both cytokine subunits [17]. Production of mIL-15/IL-15Rα complex from J100D infected cells further demonstrates that oHSV is a robust platform for transgenic expression. oHSV allows production of transgenic heterodimeric protein complexes with component genes encoded separately in the viral genome.

 Incorporation of mIL-15Rα in J100D greatly increased the production of mIL-15 as compared to J100 (Figure 3A). This is likely due to increased stability of IL-15 when in complex with IL-15Rα [32,33]. Physiologic production of IL-15 is tightly controlled. One mechanism of regulation is through signal peptides that govern trafficking of IL-15. The IL-15 long signal peptide (LSP) has been implicated in posttranslational regulation of IL-15 secretion [62]. Sequencing verified the mIL-15 gene encoded by J100 and J100D contains the mIL-15 LSP. As IL-15 is a potent proinflammatory cytokine, this LSP has been demonstrated to provide a method of limiting IL-15 production via intracellular retention and degradation [63]. Removal of the LSP or replacement with other signal peptides greatly increases IL-15 production and secretion [62,64]. An alternate method of stabilizing LSP-IL-15 is co-expression of IL-15Rα [32]. Considering the mIL-15 genes encoded by J100 and J100D are identical by sequencing, the data in Figure 3A suggests mIL-15Rα co-expressed in J100D stabilized the mIL-15 produced from this virus.

The detection of mIL-15/IL-15Rα complex in the supernates from J100D-infected cells (Figure 3B, C) suggests that the proteins were co-processed and released by mechanisms regulating IL-15 production. Production of soluble mIL-15/IL-15Rα complex could be attributable to release following oHSV replication and lysis of infected cells. However, the concentration of mIL-15/IL-15Rα complex in the supernates of J100D infected Neuro-2a cells was no different in the presence or absence of the HSV-1 replication inhibitor acyclovir (Figure S1). Additionally, the detection of mIL-15/IL-15Rα complex in the supernates of J100D-infected GL261 and CT-2A cells known to be resistant to oHSV lytic replication further argues that release of the complex was not secondary to cell lysis (Figure 3C). Although lytic replication could result in some mIL-15/IL-15Rα release into supernates, this is likely not the primary mechanism for the observed soluble mIL-15/IL-15Rα from Neuro-2a, GL261, and CT-2A cells. As increased production of soluble mIL-15/IL-15Rα complex also correlated with increased MOI, together these data indicate that production of soluble mIL-15/IL-15Rα complex is dependent on initial gene dosage at infection but does not require viral replication for continued production.

The bioactivity of soluble mIL-15/IL-15Rα produced from J100D-infected cells was demonstrated using NK cell proliferation and NK cell mediated viability reduction assays. The soluble mIL-15/IL-15Rα complex promoted the survival and stimulated the proliferation of enriched splenic NK cells with potency similar to recombinant mIL-15/IL-15Rα (Figure 4). The complex also stimulated enriched NK cells to reduce the viability of syngeneic glioma cells after 72-hour co-culture (Figure 5). In contrast, supernates derived from mock or J100 infected cells did not produce these effects from enriched NK cells. It is thus unlikely that a component of the culture media or an unknown cell or virally produced factor was responsible. Given the complexity of mIL-15 and mIL-15Rα association and trafficking, it is notable that bioactive mIL-15/IL-15Rα was produced in the presence of virally induced cellular stress. Production of bioactive mIL-15/IL-15Rα provides a foundation for future investigation with this oHSV.

To our knowledge, the studies including CT-2A cells are the first to characterize the susceptibility of this cell line to oHSV replication and direct cytotoxicity. CT-2A cells were previously utilized to investigate imaging techniques of murine brain tumors following oHSV treatment, yet no *in vitro* analyses of oHSV effects on CT-2A cells were reported [65]. CT-2A cells are a murine astrocytoma cell line derived from C57Bl/6 mice. If implanted intracerebrally, the developing CT-2A tumor models high-grade malignant glioma [50,66]. GL261 cells are also derived from C57Bl/6 mice and model high-grade malignant glioma [67]. However given that this cell line is resistant to oHSV replication and direct cytotoxicity, it is less attractive as a model for determining efficacy of novel oHSV therapies. *In vitro* studies presented here demonstrated that CT-2A cells infected with J100D produced greater amounts of mIL-15/IL-15Rα complex as compared to J100D-infected GL261 cells (Figure 3C). In addition, CT-2A cells were more susceptible to killing by oHSV than GL261 cells. The difference in susceptibility between these two cell lines is not attributable to a complete inability of oHSV to infect GL261 cells; mIL-15/IL-15Rα production from J100D infected GL261 cells was MOI dependent and increased over time (Figure 3C). Taken together, these data suggest that CT-2A cells are more susceptible to infection with oHSV. Additionally, the intracellular environment of CT-2A cells is either more permissive to viral protein production, or less susceptible to anti-viral interferon production following infection. Although CT-2A cells are poorly permissive to oHSV replication, the susceptibility to direct oHSV cytotoxicity, as well as the relatively higher production of transgenic proteins as compared to GL261 cells, argues that this cell line may be a better model for assessing malignant glioma therapy utilizing novel oHSV.

The NK cell-mediated viability reduction assay confirms that GL261 cells are susceptible to killing by NK cells. To our knowledge, this is also the first report of CT-2A susceptibility to NK killing. GL261 cells are frequently utilized to investigate immunotherapy approaches against malignant glioma, and killing of GL261 cells by NK cells has been demonstrated [67,68]. 4C8 cells have also previously been reported susceptible to NK killing [69]. However, killing of CT-2A cells has not been previously investigated. Although the assay provides evidence that CT-2A cells are susceptible to NK killing, the analysis is limited. It is unknown if killing was directly mediated or occurred indirectly through soluble factors produced by enriched NK cells. Additional studies are needed to further define the mechanism of NK cell-mediated killing of CT-2A cells.

The absence of neuropathologic signs in mice administered J100D is similar to that of other previously constructed γ_1_34.5-deleted oHSVs expressing cytokines [70]. As soluble mIL-15/IL-15Rα was detected from murine neuroblastoma (Neuro-2a) and glioma (GL261, CT-2A) cell lines following J100D infection, this virus can be utilized to study the combined effects of oHSV and mIL-15/IL-15Rα in multiple tumor models.

An important point to consider in future studies is the anti-viral effect of IL-15. Following infection of PBMCs with HSV-1, IL-15 production and release was responsible for stimulating cytotoxicity against infected cells and reducing viral replication [71,72]. In a separate study, NK killing of oHSV infected cells *in vitro* was enhanced by IL-15 [69]. IL-15 expression has been documented to increase following oHSV administration to glioma-bearing mice and rats, and may contribute to restricting oHSV replication and thus efficacy [69,73-76]. Although an experimental link between IL-15 and oHSV clearance has not been tested *in vivo*, these studies suggest IL-15 could diminish the efficacy of oHSV therapy by stimulating anti-virus immune responses. Production of IL-15 from oHSV could be self-limiting in certain tumor models. However, IL-15 produced in the tumor microenvironment can stimulate anti-tumor immune responses that improve survival [43,44]. The extent to which immune responses elicited by oHSV-produced IL-15 provide survival benefit or promote viral clearance is yet to be determined. 

In conclusion, these data further demonstrate the robust utility of oHSV for the production of immunomodulatory molecules in oncolytic virotherapy, as well as introduce a novel oHSV producing mIL-15/IL-15Rα suitable for investigation in multiple cancer models. Continuing studies are warranted to investigate anti-tumor and anti-virus immune responses elicited by oHSV production of mIL-15/IL-15Rα *in vivo*. 

## Supporting Information

Figure S1 **Acyclovir limits J100D replication but not mIL-15/IL-15Rα production.**
(TIF)

Figure S2 **Enrichment of NK cells prior to bioactivity assays.**
(TIF)
