# Supplementary figures and images for: Production of Bioactive Soluble Interleukin-15 in Complex with Interleukin-15 Receptor Alpha from a Conditionally-Replicating Oncolytic HSV-1
Source: PLoS One. 2013 Nov 27;8(11):e81768. doi: 10.1371/journal.pone.0081768 (PMC3842420; doi:10.1371/journal.pone.0081768)

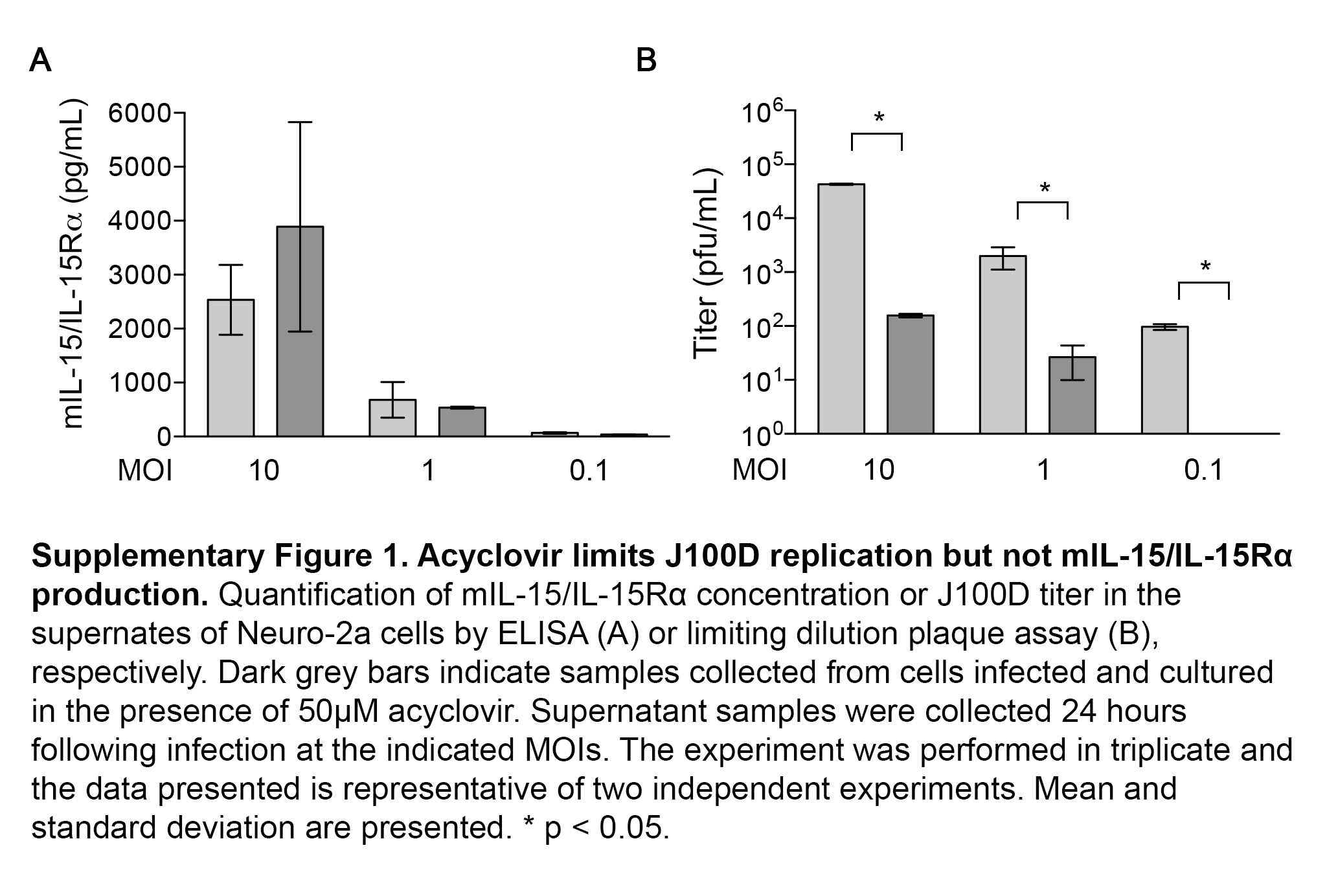

Supplement: Figure S1 — Acyclovir limits J100D replication but not mIL-15/IL-15Rα production. (TIF) [file pone.0081768.s001.tif]

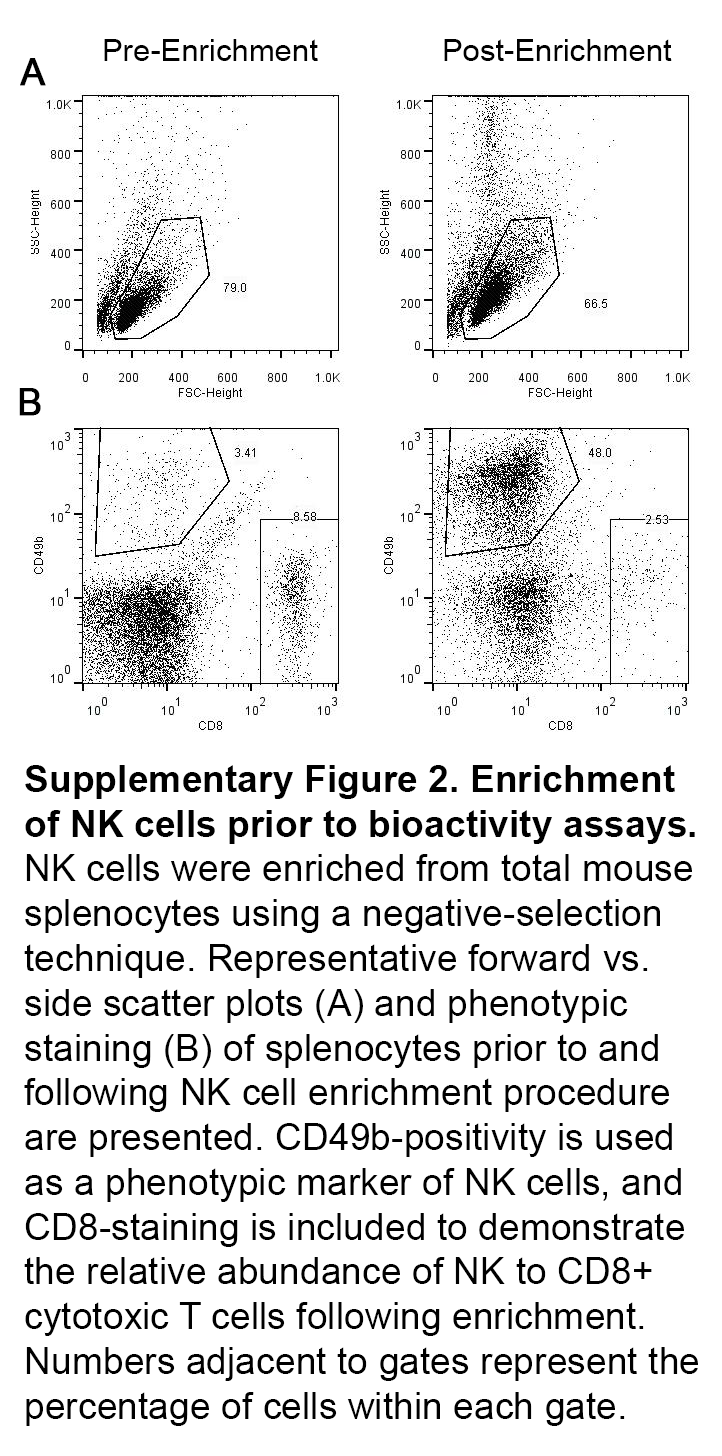

Supplement: Figure S2 — Enrichment of NK cells prior to bioactivity assays. (TIF) [file pone.0081768.s002.tif]
